# The visceral fat area to leg muscle mass ratio is significantly associated with the risk of hyperuricemia among women: a cross-sectional study

**DOI:** 10.1186/s13293-021-00360-9

**Published:** 2021-01-29

**Authors:** Xiao-He Wang, Wei-Ran Jiang, Min-Ying Zhang, Ying-Xin Shi, Yun-Ping Ji, Chun-Jun Li, Jing-Na Lin

**Affiliations:** 1 Department of Endocrinology, Health Management Center, Tianjin Union Medical Center, Nankai University Affiliated Hospital, 190 of Jieyuan Road, Hongqiao District, Tianjin, 300121 China; 2 College of Medicine, Nankai University, Tianjin, China; 3 Orofacial Pain and TMJ Disorders, Eastman Institute for Oral Health, University of Rochester, New York, NY USA

**Keywords:** Body composition, Visceral fat area, Skeletal muscle mass, Metabolic diseases, Hyperuricemia, Chinese adults

## Abstract

**Background:**

A significant positive association was found in previous studies among obesity, visceral fat accumulation, and hyperuricemia. The purpose of this study was to explore the association between the ratio of visceral fat area to leg muscle mass (VFA-to-LMM) and hyperuricemia, and verify the role of gender differences in the association.

**Methods:**

A total of 3393 (43.3% are men) participants from Tianjin Union Medical Center-Health Management Center were recruited for this cross-sectional study. The VFA-to-LMM ratio was used as the independent variable. Hyperuricemia, a serum uric acid level ≥ 416 μmol/L in men and in menopausal women and ≥ 357 μmol/L in premenopausal women, was used as the dependent variable. Multiple logistic regression analysis was used to estimate the odds ratio and the 95% confidence interval between the VFA-to-LMM ratio and hyperuricemia.

**Results:**

The overall prevalence of hyperuricemia was 14.8% (8.9% in women, and 22.5% in men). After adjustment by age, smoking status (for males), menopause status (for females), drinking status, exercise frequency, blood pressure, alanine aminotransferase, fasting plasma glucose, triglycerides, low-density lipoprotein cholesterol, high-density lipoprotein cholesterol, creatinine, and history of diseases, a strong positive association was found between the VFA-to-LMM ratio and hyperuricemia in both men (4th vs. 1st quartile 1.60, 95%CI: 1.03–2.49) and women (4th vs. 1st quartile 5.22, 95%CI: 2.44–12.56). After additional adjustment by BMI, there was still a significant positive association in women (4th vs. 1st quartile 2.57, 95%CI: 1.06–6.77). The results of subgroup analysis showed that pre-menopausal women (4th vs. 1st quartile OR: 3.61) have a higher risk of hyperuricemia than postmenopausal women (4th vs. 1st quartile OR: 1.94) with the increase of the VFA-to-LMM ratio. Besides, the interaction analysis results showed the highest risk of hyperuricemia when VFA and LMM were both in the highest quantile (OR: 11.50; 95% CI: 4.86–31.98).

**Conclusion:**

The VFA-to-LMM ratio was positively associated with the risk of hyperuricemia in women after adjustment by confounders. Pre-menopausal women have a higher risk of hyperuricemia than postmenopausal women with the increase of the VFA-to-LMM ratio. In addition, the highest risk of hyperuricemia was demonstrated when both VFA and LMM were at the highest quartile.

## Introduction

According to the National Health and Nutrition Examination Survey [1], the prevalence rates of hyperuricemia were 20.2% for men and 20.0% for women during 2007–2016, and the rates did not show any decline in the decade. Compared with the high prevalence of hyperuricemia in the USA, although China has a relatively low prevalence of hyperuricemia (19.4% in men and 7.9% in women) [2], it is still rising according to the latest data [3]. Hyperuricemia, a metabolic disease, is closely related to the inflammatory response and the disorder of glucose and lipid metabolism [4], which plays an important role in the formation of metabolic syndrome [5]. In addition, hyperuricemia, inflammation, and oxidative stress can accelerate the process of endothelial dysfunction, which is the pathophysiological basis of metabolic syndrome [6]. Moreover, hyperuricemia has also been shown to be significantly associated with lifestyle-related chronic diseases, such as hypertension, diabetes [7], non-alcoholic fatty liver disease (NAFLD) [8], and cancer. In addition, a meta-analysis based on cohort studies also confirmed that hyperuricemia was associated with high cancer incidence and mortality [9].

Considering the serious health outcomes caused by hyperuricemia, plenty of researchers have started to study its risk factors. A large population-based study confirmed that four modifiable risk factors, including the body mass index (BMI), alcohol use, diuretic use, and Dietary Approaches to Stop Hypertension diet, are independently associated with the development of hyperuricemia [10]. Among these modifiable risk factors, BMI is a comprehensive indicator that could indirectly reflect the overall condition of diet, physical activity, and metabolism. There is a complex correlation between obesity and hyperuricemia. The former can cause hyperuricemia by increasing uric acid synthesis and inhibiting its excretion, and (in turn) increased uric acid levels can promote the development of obesity by accelerating body fat accumulation (especially visceral fat) [11]. However, BMI fails to reflect the metabolic differences between men and women, such as fat distribution and skeletal muscle mass (SMM). In fact, studies have proved that there was a positive association between visceral fat and hyperuricemia [12–14], and a significant inverse association between serum uric acid (SUA) levels and SMM [15, 16]. Furthermore, studies have shown that leg composition was associated with some metabolic diseases [17, 18]. The thigh circumference which indirectly reflects leg composition has also been confirmed to be associated with various metabolic diseases, such as insulin resistance (IR), carotid atherosclerosis [19], and type-2 diabetes [20]. One explanation is that decreased SMM and fat mass in the lower limbs may be associated with disorders of glucose and lipid metabolism [21], which is closely related to hyperuricemia [22, 23].

Based on the previous studies, it can be speculated that the visceral fat area (VFA) and the leg muscle mass (LMM) seemed to have played a key role in the pathogenesis of hyperuricemia, and the combination of the two indicators would be a potential predictor of hyperuricemia. Therefore, a cross-sectional study was designed in this paper to explore the association between the VFA-to-LMM ratio and hyperuricemia, and to verify the role of gender differences in the association, so as to find a clinically feasible and gender-sensitive predictor for the risk of hyperuricemia.

## Methods

### Participants

The target population for the study was those who visited Tianjin Union Medical Center-Health Management Center for an annual physical examination from September 2019 to December 2019.

**Fig. 1:**
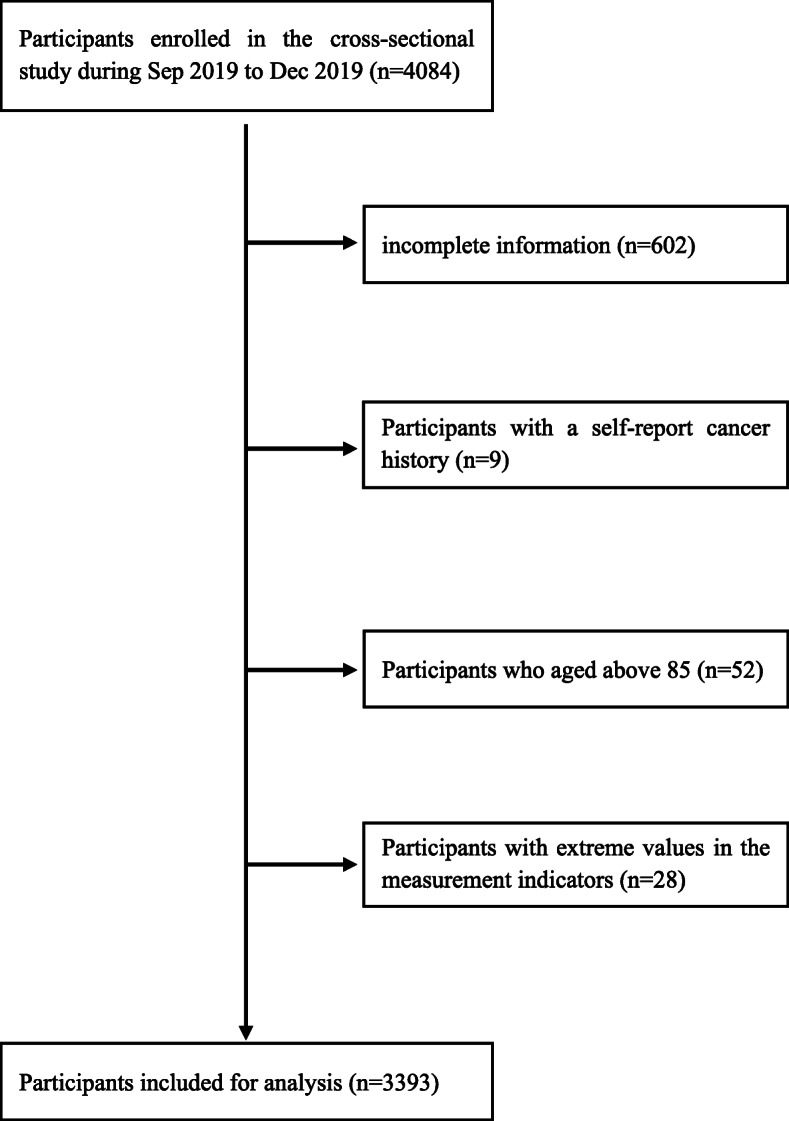
Flowchart showing the selection of the studied population

Before body composition examination, participants were interviewed and those who had recently taken specific drugs (diuretics, hypoglycemic agents, aspirin, vitamin C, etc.) were excluded because these drugs might affect uric acid metabolism. A total of 4084 eligible adults were enrolled in the study, among which 602 were excluded because they did not provide complete information, including physical examinations, biochemical analysis, questionnaires, and body composition, 9 were excluded because they had a history of cancer, and 52 were excluded because they were above 85 years old, as the association between body composition and hyperuricemia due to stability of physiological indicators or serious declination of health status might be affected. Besides, the participants (*n* = 28) who have extreme values in the measurement indicators were also excluded. Finally, 3393 (83% of those eligible) subjects had valid body composition data and covariate information required for the cross-sectional study (Fig. 1).

### Measurement of body composition

Body composition was measured by multielectrode bioelectrical impedance analyzer (Inbody 770, Biospace Inc., Korea). According to the manufacturer’s guidelines, subjects were required to fast overnight and wipe the bottom of their feet with clean water before taking measurements. They were instructed to stand barefoot on the base components with light clothing and grasp the handles of the instrument to ensure full contact with a total of 8 electrodes. Then they were asked to slightly abduct their arms and maintain the posture during the assessment. The entire measurement process took about 3–4 min, and the data were automatically saved in the computer after the measurement was completed. Based on the purpose of this study, the VFA-to-LMM ratio was selected as the independent variable.

### Covariables

Weight and height were measured by an automatic height and weight instrument (DST-600, DONGHUAYUAN, China), and participants were required to stay barefooted with light clothing during the measurement. BMI was calculated as weight in kilograms divided by the square of height in meters. All measurements are carried out in strict accordance with the national standards.

Blood pressure was measured using an automatic electronic blood pressure monitor (AC-05C, Ling Qian, China) after a 10-min rest. Each person was measured three times and the average value was taken. Hypertension is defined as systolic blood pressure (SBP) ≥ 140 mmHg and/or diastolic blood pressure (DBP) ≥ 90 mmHg or with a history of hypertension. Ultrasound liver examination was performed by an experienced ultrasound physician using an ultrasound diagnosis system (Phoenix, Philips and Neusoft Medical Systems Co., Ltd., China). The diagnosis of NAFLD was based on imaging diagnostic criteria [24]: (1) the near-field echo of the liver is diffusely enhanced (bright liver), and liver echogenicity is greater than that of kidney or spleen; (2) vascular blurring; and (3) deep attenuation of ultrasound signal. Blood biochemical analysis was performed using an automatic biochemical analyzer (TBA-120FR, Toshiba, Japan), and participants were required to fast overnight (only allowed to drink water), and venous blood was collected in a fasting state. The main indicators of blood biochemical analysis include alanine aminotransferase (ALT), fasting plasma glucose (FPG), total cholesterol (TC), triglycerides (TG), low-density lipoprotein (LDL-C), high-density lipoprotein (HDL-C) cholesterol, and serum uric acid. Diabetes is defined as FPG ≥ 7.0 or with a history of diabetes. Dyslipidemia is defined as TC ≥ 6.2 mmol/L or TG ≥ 2.3 mmol/L or LDL-C ≥ 4.1 mmol/L or HDL-C < 1.0 mmol/L according to the *Chinese Guidelines for the Management of Dyslipidemia in Adults* (*2016*) [25]. Hyperuricemia is defined as a serum uric acid level ≥ 416 μmol/L in men and menopausal women, and ≥ 357 μmol/L in premenopausal women.

The socio-demographic variables, such as gender, age, and menopause status, were collected through questionnaires. Information on lifestyles, including exercise frequency (“never,” “occasional,” and “regularly”), smoking status (“smoker,” “Ex-smoker,” and “Non-smoker”), drinking status (“drinker,” and “ex-drinker,” and “non-drinker”), and sedentary status (> 6 h/day), was also obtained in questionnaires. As for the history of diseases (including diabetes, hypertension, coronary heart disease, and cancer), participants were required to answer a “yes” or “no”.

### Statistical analysis

The characteristics of participants were presented as the means ± standard deviation or the median (interquartile range) for continuous variables and percentages for categorical variables. Statistical differences between groups were examined through analysis of variance for continuous variables and Chi-square test for categorical variables. Multiple logistic regression analysis was used to estimate the odds ratios (ORs) and 95% confidence intervals (CIs) between the VFA-to-LMM ratio and hyperuricemia. For further analysis, we fitted four models and performed a quartile conversion of the independent variable. Model 1 was adjusted by age and model 2 was adjusted by age, smoking status (for males), menopause status (for females), drinking status, exercise frequency and history of diseases. Model 3 was additionally adjusted by SBP, DBP, ALT, FPG, TC, TG, LDL-C, HDL-C, and creatinine. Model 4 was additionally adjusted by BMI based on model 3. All statistical analyses were performed with SAS 9.4 for Windows (SAS Institute, Cary, NC, USA). *P* values were two-tailed, and the differences were considered to be significant when *P* < 0.05.

## Results

### Characteristics of participants

**Fig. 2:**
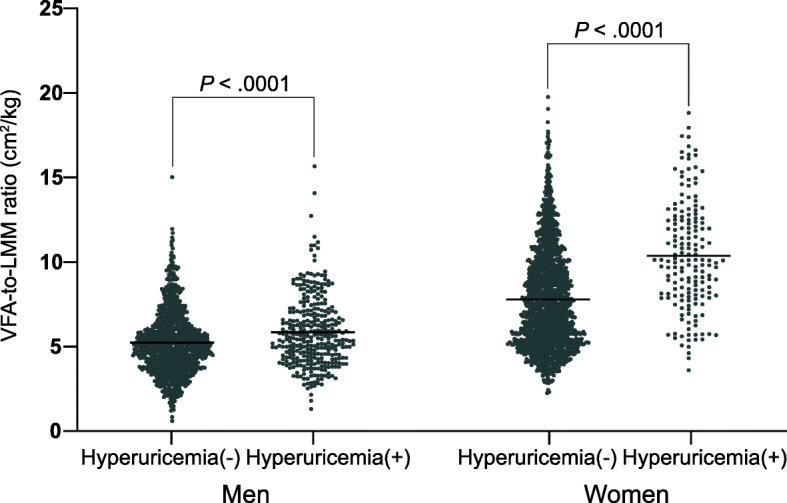
The differences in the VFA-to-LMM ratio between hyperuricemia and non-hyperuricemia among men and women. *VFA* visceral fat area, *LMM* leg muscle mass

The characteristics of participants by gender and hyperuricemia status are listed in Table 1. The overall prevalence of hyperuricemia was 14.8% (22.5% in men and 8.9% in women), and the average age of participants was 45.9 years. Analysis of the differences between the hyperuricemia group and non-hyperuricemia group indicated that there were significant differences in almost all indicators in both men and women, except for smoking, drinking, exercise, and history of diseases. Indicators, such as SBP, FPG, hypertension, and diabetes, were observed to have significant differences only in women, and indicators, such as LMM and regular exercise, were discovered to have significant differences only in men. The differences in the VFA-to-LMM ratio between hyperuricemia and non-hyperuricemia were shown in Fig. 2. According to this figure, there were significant differences in the VFA-to-LMM ratios not only between hyperuricemia and non-hyperuricemia but also between men and women (*P* < .0001).

**Table 1:** Characteristics of the study population according to sex and hyperuricemia status

Characteristics	Men (*n* = 1469)		*P*^a^	Women (*n* = 1924)		*P*
	Non-hyperuricemia (*n* = 1139)	Hyperuricemia (*n* = 330)		Non-hyperuricemia (*n* = 1753)	Hyperuricemia (*n* = 171)	
Age, years	47.9 ± 15.7	44.4 ± 14.6	0.0002	44.0 ± 14.5	54.4 ± 16.1	< .0001
BMI, kg/m^2^	25.4 ± 3.2	27.0 ± 3.5	< .0001	22.8 ± 3.2	26.0 ± 3.9	< .0001
SBP, mmHg	127.0 ± 16.8	127.8 ± 15.4	0.4377	117.3 ± 18.1	129.8 ± 19.3	< .0001
DBP, mmHg	80.2 ± 10.0	83.4 ± 10.0	< .0001	75.4 ± 9.7	79.1 ± 11.5	< .0001
VFA, cm^2^	90.7 ± 32.8	104.9 ± 36.6	< .0001	95.1 ± 35.4	129.1 ± 41.0	< .0001
LMM, kg	17.4 ± 2.2	18.0 ± 2.2	< .0001	12.3 ± 1.6	12.5 ± 1.8	0.1409
ALT, units/L	21.6 (15.3, 31.6)^b^	27.3 (18.8, 45.5)	< .0001	13.8 (10.0, 19.6)	18.3 (13.6, 26.0)	< .0001
FPG, mmol/L	5.3 (4.9, 5.9)	5.2 (5.0, 5.7)	0.3329	5.1 (4.8, 5.4)	5.6 (5.1, 6.5)	< .0001
TC, mmol/L	5.0 ± 0.9	5.2 ± 0.9	< .0001	5.1 ± 1.0	5.8 ± 1.2	< .0001
TG, mmol/L	1.3 (0.9, 1.8)	1.7 (1.2, 2.3)	< .0001	1.0 (0.7, 1.4)	1.6 (1.1, 2.2)	< .0001
LDL-C, mmol/L	2.6 ± 0.5	2.8 ± 0.5	< .0001	2.7 ± 0.6	3.1 ± 0.7	< .0001
HDL-C, mmol/L	1.3 ± 0.2	1.3 ± 0.2	< .0001	1.6 ± 0.3	1.5 ± 0.3	< .0001
SUA, μmol/L	336.0 ± 50.3	468.7 ± 47.1	< .0001	260.4 ± 45.8	395.3 ± 38.0	< .0001
Creatinine, μmol/L	74.7 ± 10.0	79.8 ± 11.7	< .0001	55.6 ± 7.7	61.5 ± 11.6	< .0001
Smoking, *n* (%)	247 (21.7)	69 (20.9)	0.7624	13 (0.7)	0 (0.0)	0.6208
Drinking, *n* (%)	459 (40.3)	129 (39.1)	0.6934	107 (6.1)	5 (2.9)	0.1275
Exercise, *n* (%)						
Never	285 (25.0)	95 (28.8)	0.1689	461 (26.3)	50 (29.2)	0.4057
Occasional	380 (33.4)	124 (37.6)	0.1557	733 (41.8)	63 (36.8)	0.2076
Regular	474 (41.6)	111 (33.6)	0.0091	559 (31.9)	58 (33.9)	0.5872
Hypertension, *n* (%)	355 (31.2)	114 (34.6)	0.2465	288 (16.4)	71 (41.5)	< .0001
Diabetes, *n* (%)	124 (10.9)	28 (8.5)	0.2071	69 (3.9)	26 (15.2)	< .0001
Dyslipidemia, *n* (%)	258 (22.7)	131 (39.7)	< .0001	316 (18.0)	76 (44.4)	< .0001
NAFLD, *n* (%)	473 (41.5)	211 (63.9)	< .0001	405 (23.1)	116 (67.8)	< .0001
Obesity, *n* (%)	232 (20.4)	122 (37.0)	< .0001	123 (7.0)	36 (21.1)	< .0001
History of diseases, *n* (%)						
Hypertension	367 (32.2)	111 (33.6)	0.6290	317 (18.1)	75 (43.9)	< .0001
Diabetes	116 (10.2)	23 (7.0)	0.0789	84 (4.8)	25 (14.6)	< .0001
CHD	68 (6.0)	18 (5.5)	0.7254	53 (3.0)	19 (11.1)	< .0001

*BMI* body mass index, *SBP* systolic blood pressure, *DBP* diastolic blood pressure, *VFA* visceral fat area, *LMM* leg muscle mass, *ALT* alanine aminotransferase, *FPG* fasting plasma glucose, *TC* total cholesterol, *TG* triglycerides, *LDL*-*C* low-density lipoprotein cholesterol, *HDL*-*C* high-density lipoprotein cholesterol, *SUA* serum uric acid, *NAFLD* non-alcoholic fatty liver diseases, *CHD* coronary heart disease

Continuous variables are expressed as means ± standard deviation (SD) and categorical variables are expressed as percentages unless otherwise indicated

^a^Analysis of variance or chi-square test where appropriate

^b^Data are expressed as medians (interquartile range)

Furthermore, the subjects were divided into four groups based on the quartiles of the VFA-to-LMM ratio, and the linear trend of each indicator (Table 2) was analyzed. As the VFA-to-LMM ratio increased, most of the indicators (BMI, FPG, TG, LDL-C, SUA) witnessed a significant linear upward trend in both men and women, and only HDL-C was found to have a downward trend.

**Table 2:** Characteristics of subjects according to quartiles of VFA-to-LMM ratio

Variables of interest	Quartiles of VFA-to-LMM ratio				*P* for Trend^a^
	Q1	Q2	Q3	Q4	
Men					
No. of subjects	367	365	369	368	
BMI, kg/m^2^	22.6 ± 2.4	25.2 ± 2.2	26.1 ± 2.1	29.0 ± 3.1	< .0001
FPG, mmol/L	5.1 (4.8, 5.5) ^c^	5.2 (4.9, 5.6)	5.4 (5.0, 5.9)	5.6 (5.1, 6.6)	< .0001
TG, mmol/L	1.0 (0.7, 1.4)	1.3 (1.0, 1.7)	1.5 (1.1, 2.1)	1.7 (1.2, 2.3)	< .0001
LDL-C, mmol/L	2.5 ± 0.5	2.7 ± 0.5	2.7 ± 0.5	2.8 ± 0.6	< .0001
HDL-C, mmol/L	1.4 ± 0.3	1.3 ± 0.2	1.3 ± 0.2	1.3 ± 0.2	< .0001
Uric acid, μmol/L	355.6 ± 68.8	363.6 ± 75.2	366.0 ± 74.4	378.0 ± 77.3	< .0001
Women					
No. of subjects	481	482	480	481	
BMI, kg/m^2^	20.0 ± 1.8	22.0 ± 2.1	23.9 ± 2.2	26.6 ± 3.1	< .0001
FPG, mmol/L	4.9 (4.7, 5.2)	5.1 (4.8, 5.4)	5.1 (4.9, 5.5)	5.4 (5.0, 6.0)	< .0001
TG, mmol/L	0.8 (0.6, 1.0)	0.9 (0.7, 1.3)	1.1 (0.8, 1.5)	1.4 (1.0, 1.9)	< .0001
LDL-C, mmol/L	2.5 ± 0.5	2.6 ± 0.5	2.8 ± 0.6	3.0 ± 0.7	< .0001
HDL-C, mmol/L	1.7 ± 0.3	1.6 ± 0.3	1.5 ± 0.3	1.5 ± 0.3	< .0001
Uric acid, μmol/L	248.8 ± 47.1	263.4 ± 52.1	276.1 ± 58.7	301.3 ± 64.8	< .0001

*BMI* body mass index, *SBP* systolic blood pressure, *DBP* diastolic blood pressure, *FPG* fasting plasma glucose, *LMM* leg muscle mass, *TC* total cholesterol, *TG* triglycerides, *LDL*-*C* low-density lipoprotein cholesterol, *HDL*-*C* high-density lipoprotein cholesterol, *VFA* visceral fat area

Continuous variables are expressed as means ± standard deviation (SD) and categorical variables are expressed as percentages unless otherwise indicated

^a^*P* for liner trends are calculated by analysis of variance (for continuous variables) or Cochran-Armitage (for categorical variables)

^b^Data are expressed as medians (interquartile range)

### VFA-to-LMM ratio and risk of hyperuricemia

The association between the VFA-to-LMM ratio and hyperuricemia was explored through multiple logistic regression models, and the results were exhibited in Table 3. After adjustment by age in model 1, a strong positive association was observed between the VFA-to-LMM ratio and hyperuricemia in both men (4th vs. 1st quartile OR: 2.73; 95% CI: 1.89–3.95) and women (4th vs. 1st quartile OR: 8.25; 95% CI: 4.05–19.14), and significant linear trends were discovered in both men (*P* < 0.0001) and women (*P* < 0.0001). After additional adjustment by smoking status (for men), menopause status (for women), drinking status, exercise frequency, SBP, DBP, ALT, FPG, TC, TG, LDL-C, HDL-C, creatinine, and history of diseases in model 3, there were still significant positive association between the VFA-to-LMM ratio and hyperuricemia in both men (4th vs. 1st quartile OR: 1.60; 95% CI: 1.03–2.49) and women (4th vs. 1st quartile OR: 5.22; 95% CI: 2.44–12.56). To further analyze whether the association was independent of general obesity, BMI was added as an adjustment factor in model 3. According to the results, there was still a significant positive association between the VFA-to-LMM ratio and hyperuricemia in women (4th vs. 1st quartile OR: 2.57; 95% CI: 1.06–6.77), while no association was observed in men (4th vs. 1st quartile OR: 0.97; 95% CI: 0.55–1.70).

**Table 3:** Odds ratio with 95% confidence interval for the association between VFA-to-LMM ratio and hyperuricemia according to sex

Quartiles of VFA-to-LMM ratio	Model 1^a^	Model 2	Model 3	Model 4
Male				
Quartile 1	1.00 (ref)	1.00 (ref)	1.00 (ref)	1.00 (ref)
Quartile 2	1.25 (0.86, 1.82)	1.21 (0.83, 1.77)	0.91 (0.60, 1.36)	0.75 (0.49, 1.15)
Quartile 3	1.58 (1.09, 2.31)	1.51 (1.03, 2.21)	1.03 (0.67, 1.57)	0.79 (0.50, 1.25)
Quartile 4	2.73 (1.89, 3.95)	2.49 (1.70, 3.65)	1.60 (1.03, 2.49)	0.97 (0.55, 1.70)
P for trend^b^	< .0001	< .0001	0.0180	0.9563
Female				
Quartile 1	1.00 (ref)	1.00 (ref)	1.00 (ref)	1.00 (ref)
Quartile 2	2.66 (1.23, 6.42)	2.58 (1.19, 6.23)	2.13 (0.96, 5.21)	1.68 (0.75, 4.17)
Quartile 3	5.20 (2.55, 12.10)	4.95 (2.41, 11.54)	3.74 (1.75, 8.95)	2.42 (1.09, 6.01)
Quartile 4	8.25 (4.05, 19.14)	7.37 (3.59, 17.24)	5.22 (2.44, 12.56)	2.57 (1.06, 6.77)
P for trend	< .0001	< .0001	< .0001	0.0454

Values are ORs (95% CIs) unless otherwise indicated. VFA, visceral fat area; LMM, leg muscle mass.

^a^Model 1: adjusted for age; model 2: adjusted for age, smoking status (for men), menopause status (for women), drinking status, exercise frequency, and history of diseases (hypertension and diabetes); model 3: additionally adjusted for systolic blood pressure, diastolic blood pressure, alanine aminotransferase, fasting plasma glucose, total cholesterol, triglycerides, low-density lipoprotein and high-density lipoprotein cholesterol, and creatinine; model 4: adjusted for model 3 + BMI

^b^*P* values for linear trends were calculated using the median value of quartiles of VFA-to-LMM ratio

**Fig. 3:**
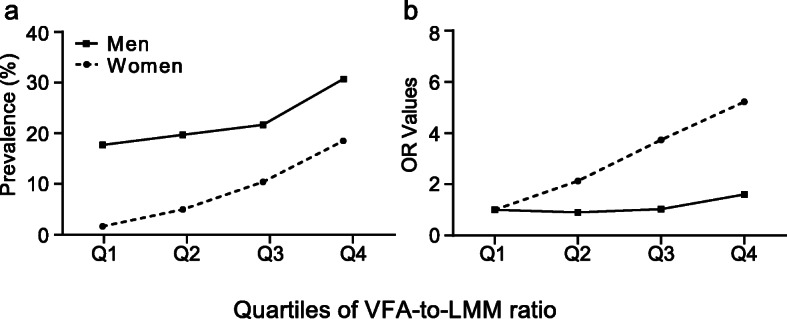
The prevalence (**a**) and odds ratio (**b**) for hyperuricemia according to quartiles of the VFA-to-LMM ratio between men and women

After that, a comparison was made on the prevalence of hyperuricemia and the odds ratios of hyperuricemia between men and women according to the quartiles of the VFA-to-LMM ratio. As shown in Fig. 3, the prevalence of hyperuricemia in women was significantly lower than that in men, but the risk of hyperuricemia in women was much higher than that in men as the VFA-to-LMM ratio increased.

### Subgroup analysis in women according to menopausal status

As shown in Table 4, the association between the VFA-to-LMM ratio and hyperuricemia in women with different menopausal status was demonstrated. According to the results, pre-menopausal women had a significantly higher risk of hyperuricemia than post-menopausal women. However, the association between the two groups was no longer significant after adjustment by BMI in model 4. It is worth noting that although the association was attenuated, the OR values of premenopausal women (4th vs. 1st quartile OR: 3.61) was still higher than that of postmenopausal women (4th vs. 1st quartile OR: 1.94), and the *P* value for trends was closer to 0.05 (*P* = 0.1503).

**Table 4:** Subgroup analysis of the association between VFA-to-LMM ratio and hyperuricemia according to menopausal status

Menopausal status	Model 1^a^	Model 2	Model 3	Model 4
Pre-menopausal				
Quartile 1	1.00 (ref)	1.00 (ref)	1.00 (ref)	1.00 (ref)
Quartile 2	3.50 (1.06, 15.75)	3.65 (1.10, 16.47)	3.63 (1.04, 17.10)	2.78 (0.79, 13.06)
Quartile 3	5.01 (1.61, 21.95)	5.30 (1.70, 23.27)	4.62 (1.36, 21.65)	2.84 (0.78, 13.74)
Quartile 4	12.04 (4.21, 50.78)	12.97 (4.50, 54.99)	8.25 (2.52, 37.99)	3.61 (0.90, 18.86)
P for trend^b^	< .0001	< .0001	0.0005	0.1503
Post-menopausal				
Quartile 1	1.00 (ref)	1.00 (ref)	1.00 (ref)	1.00 (ref)
Quartile 2	2.52 (1.29, 5.19)	2.50 (1.27, 5.18)	2.18 (1.05, 4.73)	1.79 (0.84, 3.96)
Quartile 3	2.36 (1.20, 4.89)	2.25 (1.14, 4.69)	1.85 (0.88, 4.03)	1.35 (0.61, 3.09)
Quartile 4	3.71 (1.95, 7.51)	3.43 (1.79, 6.99)	3.33 (1.64, 7.13)	1.94 (0.80, 4.81)
P for trend	0.0003	0.0010	0.0029	0.3130

Values are ORs (95% CIs) unless otherwise indicated. *VFA* visceral fat area, *LMM* leg muscle mass

^a^Model 1: adjusted for age; model 2: adjusted for age, drinking status, exercise frequency, and history of diseases (hypertension and diabetes); model 3: additionally adjusted for systolic blood pressure, diastolic blood pressure, alanine aminotransferase, fasting plasma glucose, total cholesterol, triglycerides, low-density lipoprotein and high-density lipoprotein cholesterol, and creatinine. model 4: adjusted for model 3 + BMI

^b^*P* values for linear trends were calculated using the median value of quartiles of VFA-to-LMM ratio

### The interaction of VFA and LMM on the risk of hyperuricemia

**Fig. 4:**
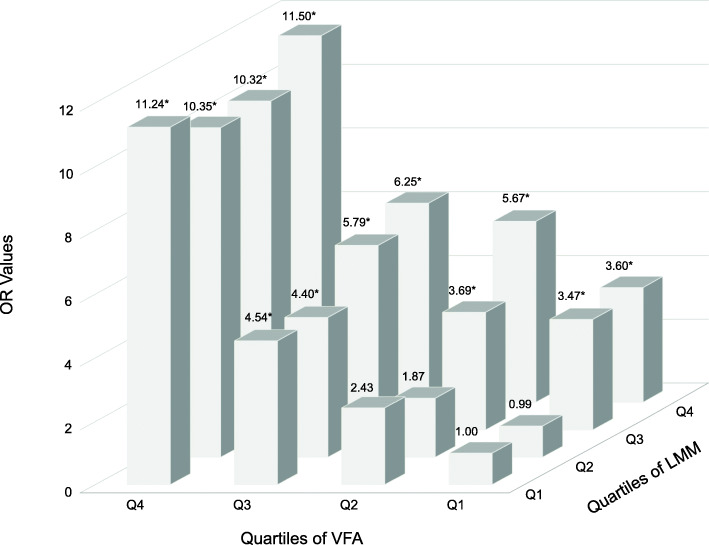
Age- and gender-adjusted odds ratios for hyperuricemia according to the quartiles of VFA and LMM. *The 95% confidence interval does not contain 1

In the further analysis, we divided the subjects into 16 groups according to the quartiles of VFA and LMM (the lowest quartile of VFA and the lowest quartile of LMM as the reference), and investigated the risk of hyperuricemia in each group after adjustment by age and gender (Fig. 4). The figure showed that the risk of hyperuricemia rose with the increasing VFA regardless of LMM, while the risk of hyperuricemia increased significantly when the LMM was in the third and highest quantiles. Remarkably, a U-shaped association was found between the risk of hyperuricemia and leg muscle mass, when VFA was in the highest quartile range, and the highest risk of hyperuricemia was discovered when VFA and LMM were both in the highest quantile (OR: 11.50; 95% CI: 4.86–31.98). The results suggested that there was an interaction between VFA and LMM, and the rising of VFA and LMM would lead to a rapid increase of hyperuricemia risks.

## Discussion

In the present study, we explored the association of the VFA-to-LMM ratio with hyperuricemia. The results showed that the VFA-to-LMM ratio was positively associated with the risk of hyperuricemia in women after adjustment by age, BMI, smoking status (for males), menopause status (for females), drinking status, exercise frequency, SBP, DBP, ALT, FPG, TC, TG, LDL-C, HDL-C, creatinine, and history of diseases. In addition, the highest risk of hyperuricemia was discovered when both VFA and LMM were at the highest quartile.

At present, the association between visceral fat mass and hyperuricemia has been confirmed by several studies. A study conducted by Huang et al. [26] found that visceral adipose accumulation was closely associated with hyperuricemia in Chinese adults. Similarly, a study from Takahashi et al. [27] confirmed the important role of visceral fat accumulation in increased serum uric acid levels in obese men. Both studies confirmed a significant association between increased visceral fat mass and hyperuricemia, and further compared the contribution of visceral and subcutaneous fat to the association. Consistent with previous studies, our results also showed a significant positive association between VFA and hyperuricemia. However, there were still limited studies on the association between skeletal muscle mass of lower limbs and hyperuricemia. In the cross-sectional study based on 7544 adults, it revealed that serum uric acid was negatively associated with skeletal muscle mass index [15]. Similarly, Tanaka et al. [28] found that a higher SUA level was associated with reduced muscle mass in men with diabetes. Conversely, several studies unveiled that a high serum uric acid level was a protective factor of muscle function [29–31], which could counteract the excessive production of free radicals that cause muscle protein damages and eventually lead to the decline of muscle mass and strength [32].

In the current study, it is found that the increase of skeletal muscle mass (or the VFA-to-SMM ratio) was not associated with hyperuricemia after adjustment by BMI, but the VFA-to-LMM ratio was independently associated with the risk of hyperuricemia. The results indicated that the relative increase in skeletal muscle mass of the lower limbs has made a great contribution to the formation of hyperuricemia. There are several indirect evidences that provide partial support. For example, in a previous study, it was found that thigh circumference, a comprehensive indicator of leg muscle mass, was strongly associated with diabetes and could be used as a good predictor [33]. In another study conducted by Min et al., it was found that there was a negative association between thigh circumference and peripheral arterial diseases when thigh circumference was less than 55 cm [34]. The preceding evidence showed that the changes of leg composition are closely related to body metabolism and health status, which may promote the formation of hyperuricemia.

The underlying mechanism of the association between body composition and hyperuricemia has not been clearly elucidated. The results might be analyzed in the following three aspects: Firstly, with the accumulation of visceral fat, some pro-inflammatory cytokines, such as IL-6, IL-8, monocyte chemoattractant protein 1 (MPC-1), and tumor necrosis factor α (TNF-α), secreted or induced by adipose tissue can lead to low-grade inflammation and oxidative stress, and further cause insulin resistance. In addition, non-esterified fatty acids (NEEAs) and resistin secreted by adipose tissue were directly related to insulin resistance [35, 36]. As the prime target of insulin, skeletal muscle was extremely important for maintaining the homeostasis of glucose and fatty acid metabolism in body. The decrease of skeletal muscle (especially leg muscle) greatly affected the metabolism of plasma fatty acid and glucose, which was further associated with insulin resistance [37]. Obviously, both visceral fat and skeletal muscle were closely associated with insulin resistance, which can lead to hyperuricemia in the following three ways: (1) IR can directly affect the reabsorption of uric acid by renal tubules and ultimately lead to the formation of hyperuricemia [38, 39]; (2) IR can indirectly cause hyperinsulinemia, which in turn provokes hyperuricemia; and (3) IR could indirectly increase the production of NADPH by promoting the lipolysis pathway, which is an important source of serum uric acid and eventually leads to hyperuricemia [40, 41]. The results showed that the increase of the VFA-to-LMM ratio led to a higher risk of hyperuricemia than the increase of VFA alone, indicating that increased LMM may play a protective role in the association, which was consistent with previous studies. Secondly, as far as we know, visceral fat accumulation is significantly associated with metabolic syndrome, while relative skeletal muscle mass was confirmed to be inversely associated with the development of metabolic syndrome [42]. Similarly, some studies have confirmed that SUA was closely related to the metabolic syndrome and may have a dual effect of cause and effect [6, 43]. It is worth noting that Kim et al. also confirmed that women with metabolic syndrome had a higher risk of hyperuricemia when they were compared with men, which was consistent with our results. Besides, some studies have shown that glucose and lipid metabolic disorder is related to the abnormal secretion of leptin, which induces oxidative stress in endothelial cells, and further leads to the increase of serum uric acid [44, 45]. Some studies have also shown that leptin, as a regulator of serum uric acid concentration, may be an intermediate factor between obesity and hyperuricemia [46]. Finally, our results showed significant gender differences, which were significant after adjustment by BMI. As we all know, there are considerable differences in both the distribution and mass of body fat and skeletal muscle between men and women due to the differences of gender hormone levels [47, 48]. Compared with women, men have a higher VFA and relatively lower SMM (or LMM), which makes the VFA-to-LMM ratio much lower than that of women. This may explain why the prevalence of hyperuricemia in men is higher than that in women, but the risk of hyperuricemia in women becomes higher as the VFA-to-LMM ratio increases. In addition, after adjustment by BMI, the association between the VFA-to-LMM ratio and hyperuricemia disappeared in men, suggesting a linear correlation between the VFA-to-LMM ratio and BMI, but for women, it is different. Therefore, the VFA-to-LMM ratio seems to have more clinical values in predicting the risk of hyperuricemia in women.

There are three advantages in the present study. First of all, as far as we know, this is the first study on the association between the VFA-to-LMM ratio and hyperuricemia. A comprehensive analysis was conducted on body composition, including VFA, LMM, and their interactions. Secondly, all measurements and statistical analysis were performed in strict accordance with the standard procedures to ensure data accuracy. Thirdly, given the significant gender differences in body composition, men and women were stratified in the analysis to ensure the reliability of the results. However, several limitations also need to be mentioned. Firstly, based on the cross-sectional data, the causality of the VFA-to-LMM ratio and hyperuricemia could not be obtained, and the potential mechanism could not be further analyzed. Secondly, although confounding factors had been adjusted as many as possible, there were still factors that were not included. However, many confounders were adjusted as possible to ensure the robustness of the results. Thirdly, due to the limitation of the sample size, further study is still needed to verify whether the association between the VFA-to-LMM ratio and hyperuricemia in subgroup analysis of women is independent of BMI.

### Perspectives and significance

According to the results, the metabolism of uric acid can be indirectly reflected in body shape, and the indicator studied in this paper can well predict the risk of hyperuricemia in women. The findings facilitated the prediction of high-risk groups of hyperuricemia by simple body composition examination or even apparent body shape. The results of subgroup analysis in the female population suggested that more attention should be paid to the changes of the VFA and LMM in pre-menopausal women. In addition, the results provided potential values for the prevention of hyperuricemia.

## Conclusion

According to the research results, the VFA-to-LMM ratio is positively associated with the risk of hyperuricemia in women after adjustment by confounding factors, and women are more sensitive than men to the risk of hyperuricemia caused by changes in body composition. In addition, pre-menopausal women have a higher risk to suffer from hyperuricemia than post-menopausal women with the increase of the VFA-to-LMM ratio. However, well-controlled prospective studies are needed to further confirm the causality between the VFA-to-LMM ratio and hyperuricemia.

## Data Availability

The datasets used and analyzed during the current study are available from the corresponding author on reasonable request.

## Abbreviations

ALT: Alanine aminotransferase
BMI: Body mass index
CI: Confidence interval
DBP: Diastolic blood pressure
FPG: Fasting plasma glucose
HDL-C: High-density lipoprotein cholesterol
IR: Insulin resistance
LDL-C: Low-density lipoprotein cholesterol
LMM: Leg muscle mass
NAFLD: Non-alcoholic fatty liver disease
OR: Odds ratio
SMM: Skeletal muscle mass
SUA: Serum uric acid
SBP: Systolic blood pressure
TC: Total cholesterol
TG: Triglycerides
VFA: Visceral fat area
