# Correction: The visceral fat area to leg muscle mass ratio is significantly associated with the risk of hyperuricemia among women: a cross-sectional study

**DOI:** 10.1186/s13293-023-00528-5

**Published:** 2023-06-29

**Authors:** Xiao-He Wang, Wei-Ran Jiang, Min-Ying Zhang, Ying-Xin Shi, Yun-Ping Ji, Chun-Jun Li, Jing-Na Lin

**Affiliations:** 1 Department of Endocrinology, Health Management Center, Tianjin Union Medical Center, Nankai University Affiliated Hospital, 190 of Jieyuan Road, Hongqiao District, Tianjin, 300121 China; 2 College of Medicine, Nankai University, Tianjin, China; 3 Orofacial Pain and TMJ Disorders, Eastman Institute for Oral Health, University of Rochester, New York, NY USA

**Correction: Biology of Sex Differences (2021) 12:17** 10.1186/s13293-021-00360-9

Following publication of the original article [1], the authors reported an error in the funding section. The corrected funding section should read as follows (change has been marked in bold):

This work was partially supported by Grants from Natural Science Foundation of Tianjin (19JCZDJC36100 to C.J.L and **18ZXDBSY00120** to J.N.L) and National Key R&D Program of China (2016YFC0900600, 2016YFC0900604 to M.Y.Z).

## References

[1] Wang X-H, Jiang W-R, Zhang M-Y, Shi Y-X, Ji Y-P, Li C-J, Lin J-N (2021). The visceral fat area to leg muscle mass ratio is significantly associated with the risk of hyperuricemia among women: a cross-sectional study. Biol Sex Differ.

